# Connectivity of corticostriatal circuits in nonmanifesting LRRK2 G2385R and R1628P carriers

**DOI:** 10.1111/cns.13933

**Published:** 2022-08-07

**Authors:** Dongling Zhang, Junye Yao, Jinghong Ma, Linlin Gao, Junyan Sun, Jiliang Fang, Hongjian He, Tao Wu

**Affiliations:** ^1^ Department of Neurology, Center for Movement Disorders, Beijing Tiantan Hospital Capital Medical University Beijing China; ^2^ China National Clinical Research Center for Neurological Diseases Beijing China; ^3^ Center for Brain Imaging Science and Technology College of Biomedical Engineering and Instrument Science Zhejiang University Hangzhou China; ^4^ Department of Neurobiology, Beijing Institute of Geriatrics Xuanwu Hospital of Capital Medical University Beijing China; ^5^ Department of Radiology, Guang'anmen Hospital China Academy of Chinese Medical Sciences Beijing China; ^6^ Parkinson's Disease Center, Beijing Institute for Brain Disorders Capital Medical University Beijing China

**Keywords:** corticostriatal circuits, functional connectivity, LRRK2, Parkinson's disease, resting‐state fMRI

## Abstract

**Background:**

Neuroimaging studies have shown that the functional connectivity (FC) of corticostriatal circuits in nonmanifesting leucine‐rich repeat kinase 2 (LRRK2) G2019S mutation carriers mirrors neural changes in idiopathic Parkinson's disease (PD). In contrast, neural network changes in LRRK2 G2385R and R1628P mutations are unclear. We aimed to investigate the FC of corticostriatal circuits in nonmanifesting LRRK2 G2385R and R1628P mutation carriers (NMCs).

**Methods:**

Twenty‐three NMCs, 28 PD patients, and 29 nonmanifesting noncarriers (NMNCs) were recruited. LRRK2 mutation analysis was performed on all participants. Clinical evaluation included MDS‐UPDRS.

**Results:**

When compared to NMNCs, NMCs showed significantly reduced FC between the caudate nucleus and superior frontal gyrus and cerebellum, and between the nucleus accumbens and parahippocampal gyrus, amygdala, and insula. We also found increased striatum‐cortical FC in NMCs.

**Conclusions:**

Although the corticostriatal circuits have characteristic changes similar to PD, the relatively intact function of the sensorimotor striatum‐cortical loop may result in less possibility of developing parkinsonian motor symptoms for the NMCs. This study helps explain why LRRK2 G2385R and R1628P mutations are risk factors rather than pathogenic mutations for PD and suggests that various LRRK2 mutations have distinct effects on neural networks.

## Graphical abstract

The relatively intact function of the sensorimotor striatum‐cortical loop may result in less possibility of developing parkinsonian motor symptoms for the nonmanifesting LRRK2 G2385R and R1628P carriers. This study helps explain why LRRK2 G2385R and R1628P mutations are risk factors rather than pathogenic mutations for PD and suggests that various LRRK2 mutations have distinct effects on neural networks.

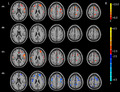

## 1 INTRODUCTION

Parkinson's disease (PD) is a progressive neurodegenerative disorder characterized by bradykinesia, resting tremor, rigidity, and postural unsteadiness. Mutations in the leucine‐rich repeat kinase (LRRK2) gene are identified as the most common cause of familial PD.^1^ LRRK2‐associated PD patients have similar clinical signs as idiopathic PD.^2^ Moreover, LRRK2 mutation carriers are at high risk for developing PD. The LRRK2 G2019S mutation has a high penetrance in Ashkenazi Jewish (25.3%) patients with PD.^3^ The risk of developing PD for LRRK2 G2019S mutation carriers was 25%–42.5% at age 80 years.^4^, ^5^ While Asians rarely have LRRK2 G2019S mutation (<0.1%).^6^ The LRRK2 G2385R and R1628P mutations are present in 4.8%–10% and 2.8%–5.2% of PD patients in Han‐Chinese population, respectively.^7^, ^8^, ^9^ However, the LRRK2 G2385R and R1628P mutations are “risk factors” rather than pathogenic mutations and are associated with a twofold increased risk of PD in populations of Han‐Chinese.^10^ The risk of nonmanifesting LRRK2 G2385R and R1628P mutation carriers (NMCs) developing PD is much lower than that of LRRK2 G2019S mutation carriers.

Brain regions densely connected at the functional level constitute global or local brain networks, therefore, investigation of the integrity of functional neural networks can help understand LRRK2 mutation‐associated alteration of brain function. Using resting‐state functional MRI (RS‐fMRI), it has been demonstrated that the right inferior parietal cortex (IPC) has reduced functional connectivity (FC) with the dorsoposterior putamen but increased FC with the ventroanterior putamen in nonmanifesting LRRK2 G2019S mutation carriers.^11^ Another RS‐fMRI study showed that nonmanifesting LRRK2 G2019S mutation carriers had reduced FC between the posterior motor part of the left striatum and the ipsilateral precuneus and superior parietal lobe.^12^ Reduced dopamine uptake in the striatum has been reported in nonmanifesting LRRK2 G2019S mutation carriers.^13^, ^14^, ^15^ A 4‐year longitudinal study found that the nonmanifesting LRRK2 G2019S mutation carriers converting to PD had lower striatal dopamine transporter binding at baseline than nonconverters.^14^ The decreased FC between the posterior putamen and cortical areas in nonmanifesting LRRK2 G2019S mutation carriers mirrors the neural changes in idiopathic PD and is likely a reflection of striatal dopamine depletion. Those findings proved that a damaged basal ganglia motor circuit has already existed in the preclinical stage of LRRK2 G2019S mutation carriers.

So far, the neural network changes in NMCs have never been investigated. The current study aimed to explore the FC of brain networks in NMCs using RS‐fMRI. We supposed that the pattern of FC of corticostriatal circuits in NMCs is different from that reported in the nonmanifesting LRRK2 G2019S mutation carriers.^11^, ^12^ It is possible that LRRK2 G2385R and R1628P mutations have less damage to the basal ganglia motor circuit than LRRK2 G2019S mutation, which might be a reason why LRRK2 G2385R and R1628P mutations are not pathogenic.

## 2 MATERIALS AND METHODS

### 2.1 Participants

This experiment was performed in accordance with the Declaration of Helsinki and was approved by the Institutional Review Board of Xuanwu Hospital. All subjects (23 NMCs, 28 PD patients, and 29 NMNCs) provided written informed consent prior to the experiment. The NMCs and NMNCs were recruited from the community cohorts of the Beijing Longitudinal Study on Aging, while PD patients were recruited from the Movement Disorders Clinic of the Xuanwu Hospital of Capital Medical University. All NMCs, PD patients, and NMNCs have received genetic screening. In the NMC group, 17 subjects had LRRK2 G2385R mutation, and six subjects had LRRK2 R1628P mutation. Genetic screening of subjects individuals in the PD and NMNC groups showed that none had LRRK2 mutations. PD patients were diagnosed according to the MDS clinical diagnostic criteria.^16^ The NMCs and PD patients were assessed with the MDS‐UPDRS (Movement Disorder Society Unified Parkinson's disease Rating Scale) by experienced neurologists. Otherwise, the Hoehn and Yahr (H&Y) disability scale was also assessed in PD patients. As the age‐dependent likelihood ratio that LRRK2 G2385R and R1628P carriers have prodromal PD remains unclear, we did not calculate the prodromal PD probability^17^ for our NMCs. The subjects with other neurological diseases or contraindications to MRI were excluded. Demographic details are shown in Table 1.

**TABLE 1:** Demographic and clinical details of the subjects

	PD (*n* = 28)	NMC (*n* = 23)	NMNC (*n* = 29)	*p* Value
Age (year)	66 **±** 5.41	69 **±** 5.04	66.34 **±** 4.43	0.065
Sex (F/M)	14/14	8/15	14/15	0.502
Disease duration (year)	4.89 **±** 3.53	—	—	—
H&Y stage	2.0	—	—	—
MDS‐UPDRS score				
Part I	7.68 **±** 6.24	3.13 **±** 2.18	—	<0.001*
Part II	9.79 **±** 6.03	0.57 **±** 1.20	—	<0.001*
Part III	27.54 **±** 12.97	2.30 **±** 2.58	—	<0.001*
Part IV	0	0	—	—

*Note*: Mean and SD are shown for continuous variables. Median is shown for H&Y stage.

Abbreviations: F, female; H&Y, Hoehn and Yahr staging; MDS‐UPDRS, Movement Disorder Society Unified Parkinson's Disease Rating Scale; M, male; NMC, nonmanifesting LRRK2 G2385R and R1628P mutation carriers; NMNC, nonmanifesting noncarriers; PD, patients with Parkinson's disease.

*p < 0.001.

### 2.2 MRI data acquisition

MRI images were acquired at a Magnetom Skyra 3T scanner (Siemens Healthcare, Erlangen, Germany). All participants were instructed to keep their eyes closed but not fall asleep during scanning. PD patients were scanned after their medication had been withdrawn for at least 12 h. RS‐fMRI data were obtained using an echo‐planar imaging (EPI) sequence with the following scanning parameter: 35 axial slices, repetition time (TR) = 2000 ms, echo time (TE) = 30 ms, flip angle = 90°, matrix = 64 × 64, voxel size = 3.438 × 3.438 × 3.6 mm^3^, field of view (FOV) = 220 × 220 mm^2^, acquisition time = 6 min. Three dimensional high resolution T1‐weighted magnetization‐prepared rapid gradient‐echo images (3D‐T1) were acquired using the following scanning parameter: TR = 2530 ms, TE = 2.98 ms, inversion time (TI) = 1100 ms, flip angle = 7°, matrix = 224 × 256, number of slices = 192, voxel size = 1 × 1 × 1 mm^3^, FOV = 256 × 256 mm^2^, acquisition time = 5 min 13 s.

### 2.3 Preprocessing of imaging data

RS‐fMRI data were preprocessed and analyzed with FSL (FMRIB Software Library v6.0, http://www.fmrib.ox.ac.uk/fsl) and DPASF5.0 (http://www.rfmri.org/dpabi). Preprocessing of RS‐fMRI data was all employed with FSL, including removing the first 10 time points, slice timing, and head motion correction. Individual 3D‐T1 brain extracted images were first registered to the standard 2 mm MNI152 space (nonlinear, >12 degrees of freedom [DOF]) using FSL's FLIRT and FNIRT tools to obtain the warping fields from individual space to standard space. Then, individual functional images were registered to 3D‐T1 images (rigid, 12 DOF) and subsequently registered to the standard space by applying the previously obtained warping fields with spline interpolation. Functional images were then smoothed with an isotropic 4 mm full width at half maximum (FWHM) Gaussian kernel, regressed head motion parameters, and low‐pass filtered (0.01–0.08 Hz). No subject was excluded on account of large head motion (more than 2.5 mm of maximal translation in any direction of *x*, *y*, or *z* or 2.5° of maximal rotation throughout the course of scanning).

### 2.4 FC analysis

As we aimed to compare the FC of corticostriatal circuits in NMCs with that reported in nonmanifesting LRRK2 G2019S mutation carriers, the regions of interest (ROIs) were defined similarly to the previous study.^11^ We focused on six distinct corticostriatal loops involving dorsoposterior, ventroposterior, dorsoanterior, and ventroanterior putamen, caudate nucleus, and nucleus accumbens in terms of functional processing and anatomical connectivity.^18^ The posterior putamen connects to the sensorimotor area, the caudate nucleus connects to the frontoparietal network, and the nucleus accumbens connects to the orbitofrontal cortex.^11^, ^19^, ^20^ We defined 12 ROIs via segmenting each subject's normalized 3D‐T1 images into bilateral putamen, bilateral caudate nucleus and nucleus accumbens (FIRST; http://www.fmrib.ox.ac.uk/fsl). We subdivided the putamen into a ventroposterior part, a dorsoposterior part, a ventroanterior part, and a dorsoanterior part using the *y* = 0 and *x* = 0 axes as the borders between the four subregions (Figure S1).^11^ The ROIs were used as the seeds for FC analysis. We obtained the reference time course of seed via calculating the average time course of each ROI. Correlation analysis was carried out via calculating the temporal correlation between the seed reference and the whole brain in a voxel‐wise manner (DPASF5.0; http://www.rfmri.org/dpabi). The individual correlation coefficient (*r*) maps were transformed into *z* map via Fisher's *Z*‐transform.

We used a one‐way analysis of covariance (ANCOVA) to analyze the difference of the FC among the NMCs, PD, and NMNCs groups, with age as a covariate of no interest. Then, the two‐tail Tukey–Kramer/hsd post hoc tests were used to compare the results between each group. The voxel *p* < 0.005 and the cluster *p* < 0.05 were considered significant thresholds, with Gaussian Random Field theory (GRF) correction. Finally, the Spearman's correlation analysis between FC value and MDS‐UPDRS scores was performed in NMC and PD groups. In addition, we analyzed the difference between G2385R and R1628P carriers by the two‐sample *t*‐test, with age and MDS‐UPDRS scores as covariates of no interest. The voxel *p* < 0.005 and the cluster *p* < 0.05 were considered significant thresholds, with GRF correction.

### 2.5 Statistical analysis

Demographic data were presented as mean ± SD. The independent two‐tail *t*‐test and Mann–Whitney test were used to compare continuous variables, and the chi‐squared test was performed for the comparison of categorical variables. The threshold for the level of significance was set at α = 0.05.

Statistical analyses were performed using SPSS Statistic software version 22 and Graphics were created using Prism version 7.0.

## 3 RESULTS

### 3.1 Demographic characteristics

All subjects completed the study, and no subject was excluded due to poor imaging data quality. There was no significant difference in terms of age and gender among the three groups. Differences were observed between the NMC and PD groups in MDS‐UPDRS I, II, and III scores (*p* < 0.001; Table 1).

### 3.2 FC in the dorsoposterior putamen

**FIGURE 1:**
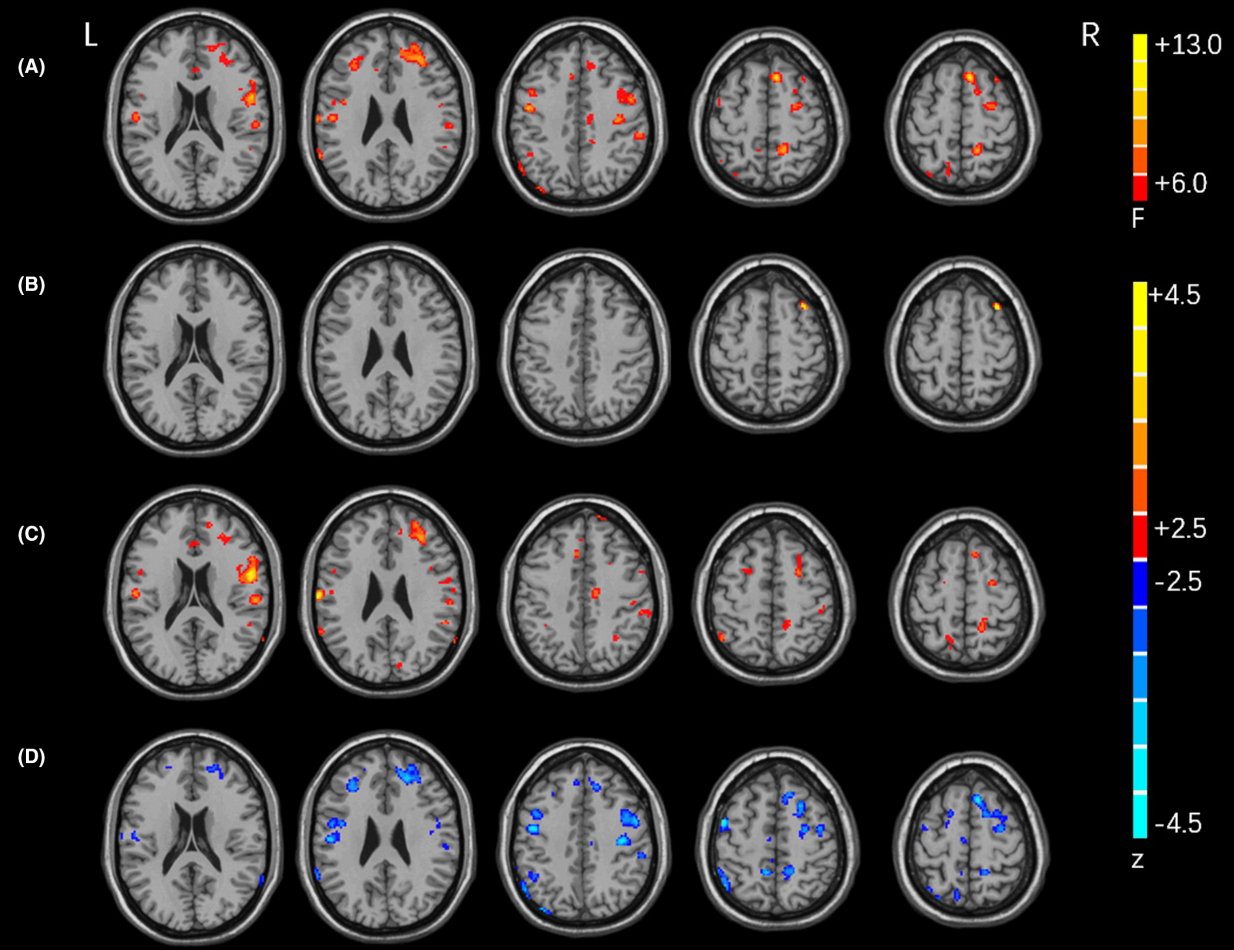
Difference of FC in the left dorsoposterior putamen between the groups. Differences of FC among NMCs, PD, and NMNC groups (A); between NMCs and NMNCs groups (B); between NMCs and PD groups (C); and between PD and NMNCs groups (D). Red/blue color indicates increased/decreased FC in NMCs compared with NMNCs, in NMCs compared with PD, or in PD compared with NMNCs, respectively. FC, functional connectivity; L, left; NMCs, nonmanifesting LRRK2 G2385R and R1628P mutation carriers; NMNCs, nonmanifesting noncarriers; PD, patients with Parkinson's disease; R, right.

There was a significant difference in the FC among the three groups between the left dorsoposterior putamen and the bilateral precentral gyrus, bilateral supplementary motor area (SMA), bilateral caudate head, bilateral putamen, and other frontal and parietal areas. NMCs had significantly increased FC with the right middle frontal gyrus compared with NMNCs and showed significantly increased FC with the right middle frontal gyrus, right precentral gyrus, and left inferior parietal gyrus compared with the PD group. PD patients had significantly reduced FC with the right middle frontal gyrus, bilateral precentral gyrus, right SMA, and left inferior parietal gyrus compared with NMNCs (Table 2 and Figure 1).

**TABLE 2:** Differences of functional connectivity in the dorsoposterior putamen between the groups

Group	Brain region	MNI Coordinates			BA	*z* value	Cluster size
		*x*	*y*	*z*			
L Dorsoposterior putamen							
NMC > NMNC	R Middle frontal gyrus	32	20	60	6	3.3095	16
NMC > PD	R Middle frontal gyrus	30	38	26	10	3.4269	146
	R Precentral gyrus	52	4	20	6	4.1936	298
	L Inferior parietal gyrus	−50	−60	52	40	3.3522	21
PD < NMNC	R Middle frontal gyrus	20	42	26	10	−3.4325	182
	R Precentral gyrus	36	−16	42	6	−3.6583	155
	L Precentral gyrus	−18	−8	68	6	−4.0701	207
	R SMA	8	22	58	6	−4.0214	481
	L Inferior parietal gyrus	−56	−44	52	40	−3.8271	185
L Dorsoposterior putamen							
NMC > NMNC	R Thalamus	16	−34	4		3.6311	71
	L Thalamus	−4	−26	6		3.1492	10
	R Middle frontal gyrus	32	20	60	6	3.3446	15
NMC > PD	R Middle frontal gyrus	24	16	46	8	3.1531	15
	L Inferior parietal gyrus	−46	−56	54	40	4.2996	323
PD < NMNC	L Superior frontal gyrus	−20	−8	68	6	−3.4088	56
	L Inferior parietal gyrus	−54	−64	42	40	−3.8549	404

*Note*: Positive/negative *z* value means increased/decreased functional connectivity between the groups.

Abbreviations: BA, Brodmann area; L, left; MNI, Montreal Neurological Institute; NMC, nonmanifesting LRRK2 G2385R and R1628P mutations carriers; NMNC, nonmanifesting noncarriers; PD, patients with Parkinson's disease; R, right; SMA, supplementary motor area.

We found significant differences in the FC among the three groups between the right dorsoposterior putamen and the left precentral gyrus, bilateral caudate head, and other frontal, parietal, temporal, and occipital areas. NMCs had increased FC with the bilateral thalamus and right middle frontal gyrus compared with NMNCs and showed increased FC with the right middle frontal gyrus and left inferior parietal gyrus compared with the PD group. PD patients had reduced FC with the left superior frontal gyrus and left inferior parietal gyrus compared with NMNCs (Table 2).

### 3.3 FC in the ventroposterior putamen

There was a significant difference in FC among the three groups between the left ventroposterior putamen and bilateral precentral gyrus, bilateral SMA, and other frontal, parietal and temporal areas. NMCs had increased FC with right middle and superior frontal gyrus compared with NMNCs and showed increased FC with right superior frontal gyrus and left medial frontal gyrus and reduced FC with right precuneus compared with the PD group. PD patients showed significantly increased FC with the right precuneus and reduced FC with the right superior, middle frontal gyrus, and right SMA compared with NMNCs (Table S1).

We found significant differences among the three groups between the right ventroposterior putamen and the left superior frontal gyrus, left caudate, left middle occipital gyrus, right fusiform gyrus, and right middle temporal gyrus. NMCs had significantly increased FC with the left superior frontal gyrus compared with NMNC and PD groups, while PD patients had significantly reduced FC with the left superior frontal gyrus compared with NMNCs (Table S1).

### 3.4 FC in the dorsoanterior putamen

There was a significant difference in FC among the three groups between the left dorsoanterior putamen and the bilateral SMA, right middle frontal gyrus, right inferior frontal gyrus, right middle temporal gyrus, left precentral gyrus, and left caudate nucleus. NMCs had increased FC with the right postcentral gyrus compared with NMNCs and showed increased FC with the right middle frontal gyrus compared with the PD group. PD patients had reduced FC with the right middle frontal gyrus and right SMA compared with NMNCs (Table S2).

We found a significant difference in FC among the three groups between the right dorsoanterior putamen and the right caudate and frontal and parietal areas. NMCs had reduced FC with the left superior frontal gyrus and increased FC with the right middle frontal gyrus compared with NMNCs and showed increased FC with the left lingual gyrus and left superior parietal gyrus compared with the PD group. PD patients had reduced FC with the left lingual gyrus, left superior parietal gyrus, and right SMA compared with NMNCs (Table S2).

### 3.5 FC in the ventroanterior putamen

We found a significant difference in FC among the three groups between the left ventroanterior putamen and the bilateral SMA, bilateral middle frontal gyrus, left medial frontal gyrus, left superior frontal and right inferior frontal gyrus. NMCs had increased FC with the right middle frontal gyrus compared with NMNCs, and showed significantly increased FC with the right middle frontal gyrus, right SMA, and left superior frontal gyrus compared with the PD group. PD patients had significantly reduced FC with the right middle frontal gyrus, right SMA, and left superior frontal gyrus compared with NMNCs (Table S3).

There was a significant difference in FC among the three groups between the right ventroanterior putamen and the bilateral inferior frontal gyrus, left medial frontal gyrus, left caudate, right inferior parietal gyrus, and right SMA. NMCs showed increased FC with the left medial frontal gyrus compared with NMNCs and PD groups. PD patients had significantly reduced FC with the left middle frontal gyrus compared with NMNCs (Table S3).

### 3.6 FC in the caudate nucleus

We found a significant difference in FC among the three groups between the left caudate nucleus and the frontal, parietal, temporal, and occipital areas, pons, midbrain, and cerebellum. NMCs showed reduced FC with the left superior frontal gyrus and increased FC with the right middle temporal gyrus, right inferior frontal gyrus, pons, and right putamen compared with NMNCs and showed increased FC with the right middle temporal gyrus, right inferior frontal gyrus, right middle cingulum, left middle occipital gyrus, and right cerebellum compared with the PD group. PD patients had reduced FC with the left superior frontal gyrus, right middle temporal gyrus, right inferior frontal gyrus, right middle cingulum, right cerebellum, and midbrain compared with NMNCs (Table S4 and Figure S2).

There was a significant difference in FC among the three groups between the right caudate nucleus and the right middle temporal gyrus, right inferior parietal gyrus, right middle frontal gyrus and left cerebellum. NMCs had reduced FC with the left cerebellum and left superior frontal gyrus compared with NMNCs and had increased FC with the left cerebellum, right middle temporal gyrus, left middle occipital gyrus, and left supramarginal gyrus compared with the PD group. PD patients had reduced FC with the left cerebellum, right middle temporal gyrus, left middle occipital gyrus, and right supramarginal gyrus compared with NMNCs (Table S4).

### 3.7 FC in the nucleus accumbens

There was a significant difference in FC among the three groups between the left nucleus accumbens and the bilateral insula, bilateral parahippocampal gyrus, left postcentral gyrus, left superior temporal gyrus, and bilateral middle cingulum. NMCs showed reduced FC with the left parahippocampal gyrus, left amygdala, left insula, and left postcentral gyrus compared with NMNCs and had increased FC with the left postcentral gyrus, right superior temporal gyrus, and right insula compared with the PD group. PD patients had reduced FC with the left Rolandic gyrus, left parahippocampal gyrus, left insula, left postcentral gyrus, right superior temporal gyrus, and right insula compared with NMNCs (Table S5 and Figure S3).

We found significant differences of FC among the three groups between the right nucleus accumbens and the left insula, bilateral parahippocampal gyrus, and right lingual gyrus. NMCs had reduced FC with the right lingual gyrus compared with NMNCs, and showed increased FC with the left caudate, right inferior frontal gyrus, and right middle temporal gyrus compared with the PD group. PD patients had reduced FC with the left putamen, right lingual gyrus, right inferior frontal gyrus, and right middle temporal gyrus compared with NMNCs (Table S5).

### 3.8 Correlation analysis

**FIGURE 2:**
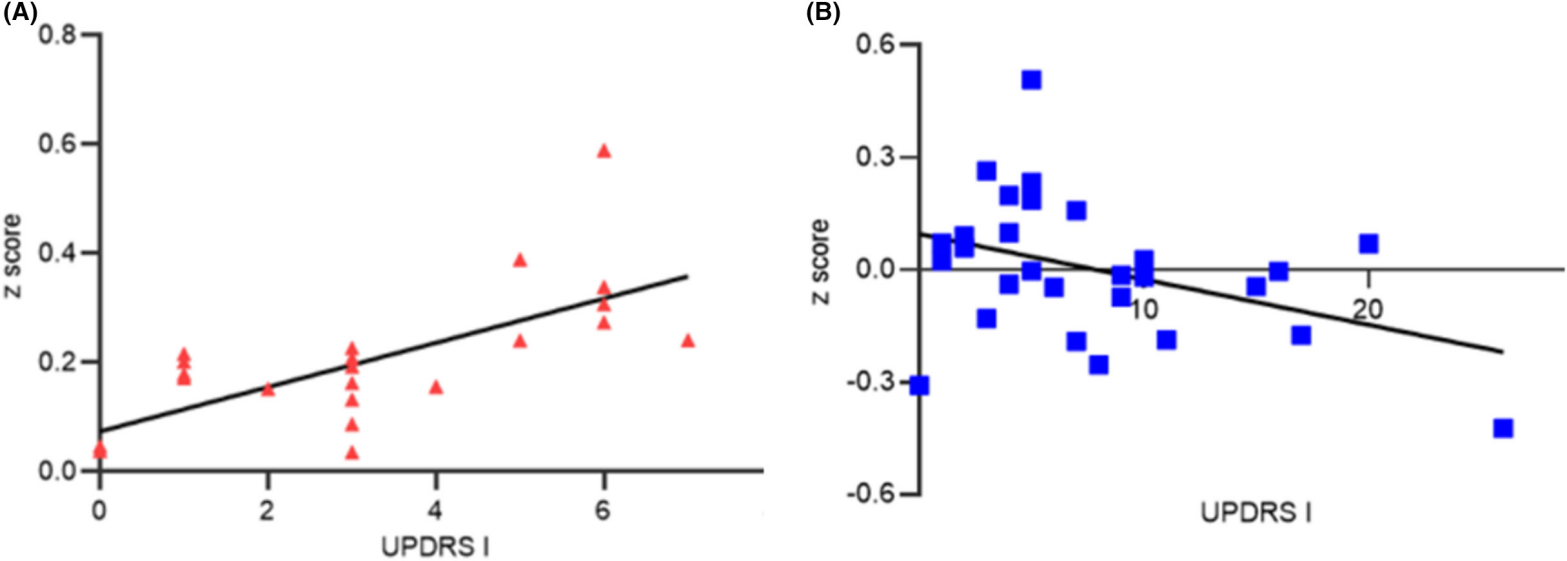
The results of correlation analysis. FC between the left caudate nucleus and right inferior frontal gyrus positively correlated with MDS‐UPDRS I scores in the NMC group (A) and negatively correlated with MDS‐UPDRS I scores in PD patients (B). NMC, nonmanifesting LRRK2 G2385R and R1628P mutations carriers; PD, patients with Parkinson's disease.

Correlation analysis showed that in NMCs, FC between the left caudate nucleus and right inferior frontal gyrus had a positive correlation with MDS‐UPDRS I scores (*r* = 0.604, *p* = 0.002). In the PD group, FC between the left caudate nucleus and right inferior frontal gyrus had a negative correlation with MDS‐UPDRS I scores (*r* = −0.403, *p* = 0.033; Figure 2).

### 3.9 Comparison between LRRK2 mutations

No significant difference was found in the comparison of FC between nonmanifesting LRRK2 G2385R and R1628P mutation in any ROIs.

## 4 DISCUSSION

The present study, for the first time, investigated the FC of corticostriatal circuits in the NMCs. We found that although the pattern of network connectivity was disrupted, NMCs did not show dysfunction of the posterior putamen‐cortical loop.

In PD patients, dopamine is significantly depleted mostly in the posterior putamen,^21^ which is a part of the sensorimotor striatum. Consistent with previous reports,^22^, ^23^ our PD patients had decreased FC between the posterior putamen and cortical motor areas, such as the SMA and precentral gyrus. The impaired motor striatum‐cortical loop is critical to some typical parkinsonian motor symptoms, e.g., bradykinesia. Nonmanifesting LRRK2 G2019S mutation carriers also have decreased posterior putamen‐cortical FC,^11^, ^12^ and have reduced dopamine uptake in the striatum,^13^, ^14^ The dysfunction of basal ganglia motor circuit provides explains why LRRK2 G2019S mutation carriers are at high risk for developing PD.

In contrast, we did not observe reduced FC between posterior putamen and cortical areas in NMCs. In PD patients with LRRK2 G2385R mutation, the dopamine uptake is similar to that in idiopathic PD,^24^ However, whether NMCs have dopamine depletion in the striatum has not been reported, and remains unclear. As the function of the posterior putamen‐cortical loop is not significantly impaired, it is possible that dopamine depletion in the sensorimotor striatum is not significant in NMCs. A relatively intact sensorimotor striatum‐cortical circuit should result in less possibility of developing parkinsonian motor deficits, which may help explain why LRRK2 G2385R and R1628P mutations are not pathogenic. A recent study showed that PD patients with LRRK2 G2385R and R1628P mutations have decreased FC between the posterior putamen and bilateral sensorimotor cortices,^25^ Thus, although not significant, some of our NMCs may have decreased FC in the sensorimotor striatum‐cortical circuit. Further longitudinal studies are needed to investigate the relationship between FC changes in the sensorimotor striatum‐cortical circuit and PD conversion in NMCs.

Our NMCs showed reduced FC between the caudate nucleus and superior frontal gyrus and cerebellum. The caudate nucleus receives massive projections from the frontal cortex. Decreased FC between the caudate nucleus and frontal cortex^26^ and cerebellum^27^ has been reported in PD patients and related to cognitive performance. In addition, we found that in NMCs, FC between the left caudate nucleus and right inferior frontal gyrus had a positive correlation with MDS‐UPDRS I scores. It has been demonstrated that some nonmotor symptoms in MDS‐UPDRS I, like cognitive impairment and autonomic dysfunction, can precede dopamine transporter deficit in nonmanifesting LRRK2 mutation carriers.^15^

NMCs also had decreased FC between the nucleus accumbens and parahippocampal gyrus, amygdala, insula, postcentral gyrus, lingual gyrus, and caudate nucleus. The nucleus accumbens is a critical node of the mesocorticolimbic system receiving projections from the amygdala, hippocampus, insular and prefrontal cortex, and plays an important role in processing reward and emotional contextual information.^28^, ^29^ In nonmanifesting LRRK2 G2019S mutation carriers, the nucleus accumbens had a decreased activation in risky anticipation while performing gambling task.^30^ The changes in the FC of the caudate nucleus and nucleus accumbens in NMCs mirror the neural changes in PD patients, which indicates that NMCs have some characteristic changes in the corticostriatal circuit similar to PD patients. Whether the impaired FC in the caudate nucleus and nucleus accumbens relating to cognitive, emotional, or other nonmotor problems in NMCs needs to be investigated in future.

NMCs also exhibited some enhanced FC in the putamen and caudate nucleus. Cortical areas showed increased connectivity with the striatum including the middle temporal gyrus (involved in the dorsal attention network, DAN), prefrontal cortex (involved in the salience network, SAL), and medial frontal gyrus (involved in the default mode network, DMN). The increased FC may reflect a functional compensation to the impaired corticostriatal circuits.^31^ It has been shown that dopamine depletion can induce new sprouting and branches of striatal dopamine axons as a compensatory response.^32^ In contrast, a recent study found that nonmanifesting LRRK2 G2019S mutation carriers had lower connectivity measures in the DMN, SAL, and DAN compared with NMNCs.^33^ Therefore, it is possible that various LRRK2 mutations not only affect corticostriatal circuits differently but also have distinct effects on cortical neural networks.

We did not find a significant difference between nonmanifesting LRRK2 G2385R and R1628P mutation, which suggests that these two mutations have a similar pattern of FC of corticostriatal circuits.

Some limitations should be addressed. First, longitudinal studies are needed to explore the dynamic alterations of neural networks in NMCs. Second, the sample size was relatively small, more NMCs will be recruited in future. Third, the nonmotor symptom was not evaluated in NMCs and PD patients.

In conclusion, although there were characteristic changes in the corticostriatal circuits similar to PD, we did not detect significantly damaged posterior putamen‐cortical circuits in NMCs. The relatively intact function of the sensorimotor striatum‐cortical loop may result in less possibility of developing parkinsonian motor symptoms. Our findings suggest that various LRRK2 mutations have distinct effects on neural networks, and may help explain why LRRK2 G2385R and R1628P mutations are risk factors rather than pathogenic mutations for PD.

## CONFLICT OF INTEREST

The authors have no conflict of interest to report.

## Supporting information


Figure S1

Figure S2

Figure S3

Tables S1

## Data Availability

The data that support the findings of this study are available from the corresponding author upon reasonable request.
