# Supplementary figures and images for: Connectivity of corticostriatal circuits in nonmanifesting LRRK2 G2385R and R1628P carriers
Source: CNS Neurosci Ther. 2022 Aug 7;28(12):2024–31. doi: 10.1111/cns.13933 (PMC9627388; doi:10.1111/cns.13933)

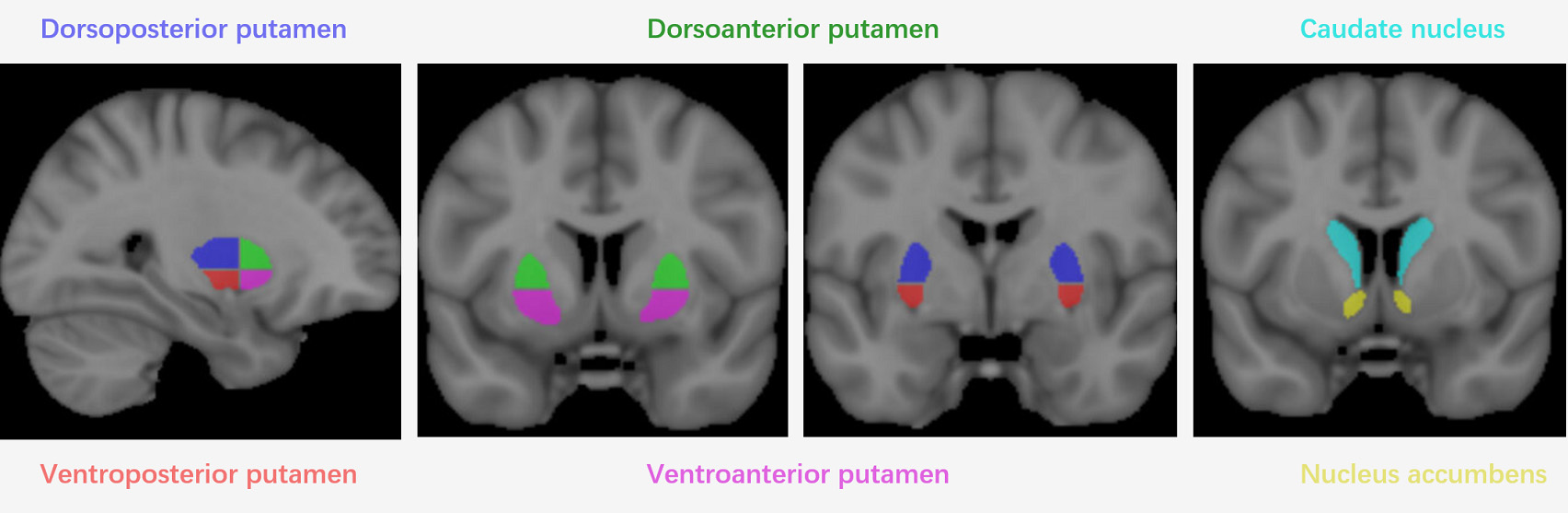

Supplement: Supplementary file 1 — Figure S1 [file CNS-28-2024-s002.jpg]

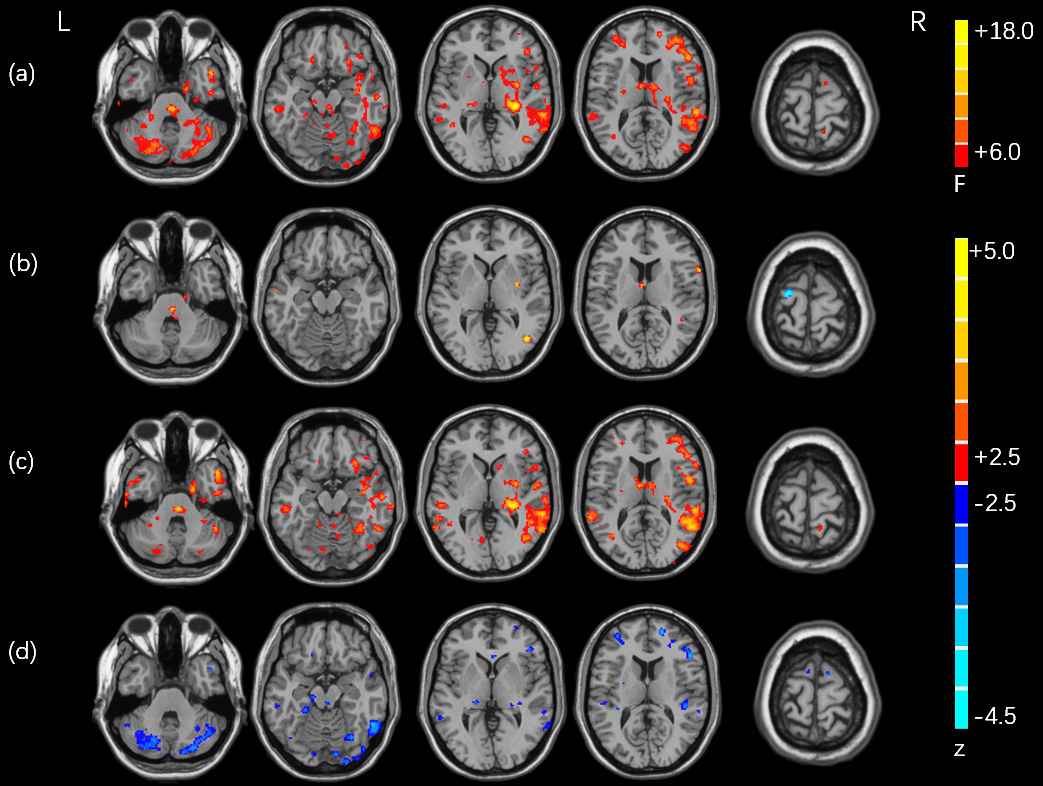

Supplement: Supplementary file 2 — Figure S2 [file CNS-28-2024-s003.jpg]

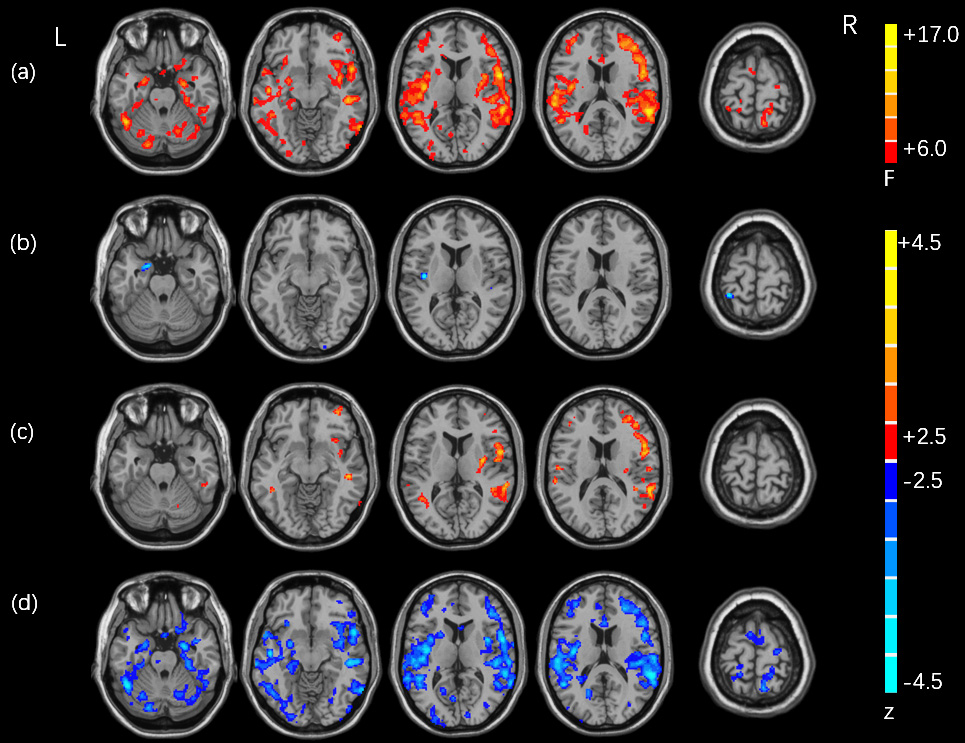

Supplement: Supplementary file 3 — Figure S3 [file CNS-28-2024-s001.jpg]
