# Supplementary material for: Connectivity of corticostriatal circuits in nonmanifesting LRRK2 G2385R and R1628P carriers
Source: CNS Neurosci Ther. 2022 Aug 7;28(12):2024–31. doi: 10.1111/cns.13933 (PMC9627388; doi:10.1111/cns.13933)
Supplement: Supplementary file 4 — Tables S1 [file CNS-28-2024-s004.docx]

**Supplementary Tables**

**Table S1.** Differences of functional connectivity in the ventroposterior putamen between the groups

| Group | Brain region | MNI Coordinates | | | BA | z value | Cluster  size |
| --- | --- | --- | --- | --- | --- | --- | --- |
|  |  | x | y | z |  |  |  |
| L Ventroposterior Putamen | |  |  |  |  |  |  |
| NMC > NMNC | R Middle Frontal Gyrus | 32 | 24 | 60 | 8 | 3.1272 | 16 |
|  | R Superior Frontal Gyrus | 20 | 48 | 50 | 8 | 3.0786 | 11 |
| NMC > PD | R Superior Frontal Gyrus | 10 | 58 | 36 | 9 | 3.752 | 180 |
|  | L Medial Frontal Gyrus | -8 | 64 | -2 | 6 | 4.1263 | 203 |
|  | L Precentral gyrus | -46 | 6 | 22 |  | 3.1524 | 17 |
| NMC < PD | R Precuneus | 26 | -46 | 6 | 7 | -3.8943 | 105 |
| PD > NMNC | R Precuneus | 24 | -44 | 16 | 7 | 3.4278 | 102 |
| PD < NMNC | R Superior Frontal Gyrus | 4 | 50 | 34 | 9 | -3.7758 | 208 |
|  | R Middle Frontal Gyrus | 40 | -4 | 54 | 6 | -3.6035 | 187 |
|  | R SMA | 8 | 20 | 58 | 6 | -3.6434 | 77 |
| R Ventroposterior Putamen | |  |  |  |  |  |  |
| NMC > NMNC | L Superior Frontal Gyrus | -12 | 66 | -8 | 10 | 3.3216 | 13 |
| NMC > PD | L Superior Frontal Gyrus | -10 | 68 | -8 | 10 | 4.2698 | 325 |
| PD < NMNC | L Superior Frontal Gyrus | -10 | 64 | 8 | 10 | -3.4831 | 19 |

Positive/negative z value means increased/decreased functional connectivity between the groups; MNI, Montreal Neurological Institute; BA, Brodmann area; L, left; R, right; PD, patients with Parkinson's disease; NMC, non-manifesting LRRK2 G2385R and R1628P mutations carriers; NMNC, non-manifesting non-carriers; SMA, supplementary motor area.

**Table S2.** Differences of functional connectivity in the dorsoanterior putamen between the groups

| Group | Brain region | MNI | | | BA | z value | Cluster size |
| --- | --- | --- | --- | --- | --- | --- | --- |
|  |  | x | y | z |  |  |  |
| L Dorsoanterior Putamen | |  |  |  |  |  |  |
| NMC > NMNC | R Postcentral Gyrus | 16 | -44 | 80 |  | 3.3445 | 11 |
| NMC > PD | R Middle Frontal Gyrus | 24 | 44 | 28 | 10 | 3.331 | 54 |
| PD < NMNC | R Middle Frontal Gyrus | 24 | 44 | 30 | 10 | -3.6418 | 92 |
|  | R SMA | 8 | 22 | 58 | 6 | -4.3674 | 97 |
| R Dorsoanterior Putamen | |  |  |  |  |  |  |
| NMC < NMNC | L Superior Frontal Gyrus | -20 | -4 | 72 | 6 | -3.3255 | 16 |
| NMC > NMNC | R Middle Frontal Gyrus | 34 | 20 | 58 | 8 | 3.403 | 19 |
| NMC > PD | L Lingual Gyrus | -6 | -66 | 4 | 30 | 3.1896 | 17 |
|  | L Superior Parietal Gyrus | 40 | -86 | 30 | 7 | 4.3326 | 85 |
| PD < NMNC | L Lingual Gyrus | -6 | -62 | 4 | 30 | -3.7181 | 55 |
|  | L Superior Parietal Gyrus | -28 | -72 | 58 | 7 | -3.9831 | 333 |

Positive/negative z value means increased/decreased functional connectivity between the groups; MNI, Montreal Neurological Institute; BA, Brodmann area; L, left; R, right; PD, patients with Parkinson's disease; NMC, non-manifesting LRRK2 G2385R and R1628P mutations carriers; NMNC, non-manifesting non-carriers; SMA, supplementary motor area.

**Table S3.** Differences of functional connectivity in the ventroanterior putamen between the groups

| Group | Brain region | MNI | | | BA | z value | Cluster size |
| --- | --- | --- | --- | --- | --- | --- | --- |
|  |  | x | y | z |  |  |  |
| L Ventroanterior Putamen | |  |  |  |  |  |  |
| NMC > NMNC | R Middle Frontal Gyrus | 32 | 22 | 58 | 6 | 3.5017 | 30 |
| NMC > PD | R Middle Frontal Gyrus | 26 | 50 | 30 | 10 | 3.2104 | 14 |
|  | R SMA | 8 | 22 | 60 | 6 | 3.5232 | 51 |
|  | L Superior Frontal Gyrus | -14 | 46 | 30 | 9 | 3.2078 | 11 |
| PD < NMNC | R Middle Frontal Gyrus | 24 | 46 | 30 | 10 | -3.5531 | 267 |
|  | R SMA | 8 | 20 | 58 | 6 | -4.1492 | 132 |
|  | L Superior Frontal Gyrus | -16 | 46 | 30 | 9 | -3.8791 | 224 |
| R Ventroanterior Putamen | |  |  |  |  |  |  |
| NMC > NMNC | L Medial Frontal Gyrus | -10 | 64 | -6 | 10 | 3.4118 | 16 |
| NMC > PD | L Medial Frontal Gyrus | -10 | 62 | -6 | 10 | 3.9373 | 333 |
| PD < NMNC | L Middle Frontal Gyrus | -24 | 42 | 22 | 10 | -3.0806 | 72 |

Positive/negative z value means increased/decreased functional connectivity between the groups; MNI, Montreal Neurological Institute; BA, Brodmann area; L, left; R, right; PD, patients with Parkinson's disease; NMC, non-manifesting LRRK2 G2385R and R1628P mutations carriers; NMNC, non-manifesting non-carriers; SMA, supplementary motor area; SMA, supplementary motor area.

**Table S4.** Differences of functional connectivity in the caudate nucleus between the groups

| Group | | Brain region | MNI | | | BA | z value | Cluster size |
| --- | --- | --- | --- | --- | --- | --- | --- | --- |
|  |  |  | x | y | z |  |  |  |
| L Caudate Nucleus |  | |  |  |  |  |  |  |
| NMC < NMNC | L Superior Frontal Gyrus | | -24 | -10 | 74 | 6 | -3.0739 | 27 |
| NMC > NMNC | R Middle Temporal Gyrus | | 44 | -68 | 4 | 37 | 3.8187 | 77 |
|  | R Inferior Frontal Gyrus | | 64 | 14 | 12 | 44 | 3.4471 | 43 |
|  | R SupraMarginal Gyrus | | 54 | -42 | 28 |  | 3.3569 | 10 |
|  | R Putamen | | 32 | -4 | 2 |  | 3.5502 | 10 |
|  | Pons | | 2 | -28 | -34 |  | 3.2985 | 50 |
| NMC > PD | R Middle Temporal Gyrus | | 50 | -30 | -10 | 40 | 5.0384 | 5980 |
|  | R Inferior Frontal Gyrus | | 26 | 26 | -14 | 47 | 3.7839 | 402 |
|  | R Middle Cingulum | | 6 | -44 | 38 | 31 | 4.0471 | 116 |
|  | L Middle Occipital Gyrus | | -28 | -84 | 40 | 19 | 4.222 | 260 |
|  | R Cerebellum Posterior Lobe | | 36 | -70 | -30 |  | 4.0258 | 109 |
| PD < NMNC | L Superior Frontal Gyrus | | -18 | 28 | -24 |  | -3.6667 | 29 |
|  | R Middle Temporal Gyrus | | 48 | -26 | -10 | 37 | -4.3631 | 160 |
|  | R Inferior Frontal Gyrus | | 50 | 28 | 12 |  | -3.8225 | 124 |
|  | R Middle Cingulum | | 4 | -44 | 36 | 31 | -3.5426 | 72 |
|  | R Cerebellum Posterior Lobe | | 34 | -70 | -30 |  | -4.6778 | 740 |
|  | Midbrain | | 2 | -26 | -16 |  | -3.1056 | 18 |
| R Caudate Nucleus | | |  |  |  |  |  |  |
| NMC < NMNC | L Cerebellum Posterior Lobe | | -36 | -63 | -32 |  | -3.5222 | 33 |
|  | L Superior Frontal Gyrus | | -20 | -4 | 72 |  | -3.032 | 14 |
| NMC > PD | L Cerebellum Anterior Lobe | | -20 | -44 | -20 |  | 3.6724 | 31 |
|  | R Middle Temporal Gyrus | | 64 | -40 | 0 | 21 | 4.4867 | 505 |
|  | L Middle Occipital Gyrus | | -28 | -84 | 40 | 19 | 4.222 | 260 |
|  | R SupraMarginal Gyrus | | 52 | -42 | 26 | 40 | 3.7353 | 203 |
| PD < NMNC | L Cerebellum Posterior Lobe | | -36 | -62 | -32 |  | -3.9653 | 247 |
|  | R Middle Temporal Gyrus | | 46 | -24 | -10 | 21 | -4.5108 | 238 |
|  | L Middle Occipital Gyrus | | -32 | -84 | 40 | 19 | -3.9782 | 92 |
|  | R SupraMarginal Gyrus | | 56 | -42 | 44 | 40 | -3.434 | 72 |

Positive/negative z value means increased/decreased functional connectivity between the groups; MNI, Montreal Neurological Institute; BA, Brodmann area; L, left; R, right; PD, patients with Parkinson's disease; NMC, non-manifesting LRRK2 G2385R and R1628P mutations carriers; NMNC, non-manifesting non-carriers.

**Table S5.** Differences of functional connectivity in the nucleus accumbens between the groups

| \| Group \| Brain region \| MNI Coordinates \| \| \| BA \| z value \| Cluster  size \| \| --- \| --- \| --- \| --- \| --- \| --- \| --- \| --- \| \| x \| y \| z \| | | | | | | | |
| --- | --- | --- | --- | --- | --- | --- | --- | --- | --- | --- | --- | --- | --- | --- | --- | --- | --- | --- |
| L Nucleus Accumbens | |  |  |  |  |  |  |
| NMC < NMNC | L ParaHippocampal Gyrus | -18 | 2 | -24 |  | -3.417 | 43 |
|  | L Amygdala |  |  |  |  |  | 29 |
|  | L Insula | -38 | -12 | 10 |  | -3.5176 | 33 |
|  | L Postcentral Gyrus | -32 | -42 | 70 |  | -3.5867 | 22 |
| NMC > PD | L Postcentral Gyrus | -56 | -14 | 16 | 43 | 3.2115 | 13 |
|  | R Superior Temporal Gyrus | 60 | -50 | 20 | 40 | 4.3848 | 1335 |
|  | R Insula | 32 | 18 | -10 | 13 | 3.0631 | 10 |
| PD < NMNC | L Rolandic Gyrus | -40 | -10 | 12 | 13 | -4.713 | 8468 |
|  | L ParaHippocampal Gyrus |  |  |  |  |  | 125 |
|  | L Insula |  |  |  |  |  | 452 |
|  | L Postcentral Gyrus |  |  |  |  |  | 254 |
|  | R Superior Temporal Gyrus | 58 | -40 | 16 | 40 | -4.8549 | 14524 |
|  | R Insula |  |  |  |  |  | 535 |
| R Nucleus Accumbens | |  |  |  |  |  |  |
| NMC < NMNC | R Lingual Gyrus | 22 | -88 | -4 |  | -3.8377 | 51 |
| NMC > PD | L Caudate | -8 | 4 | 12 |  | 3.8683 | 44 |
|  | R Inferior Frontal Gyrus | 46 | 38 | 6 | 10 | 3.6522 | 246 |
|  | R Middle Temporal Gyrus | 56 | -52 | 0 | 22 | 3.778 | 178 |
| PD < NMNC | L Putamen | -26 | 0 | 6 |  | -3.206 | 45 |
|  | R Lingual Gyrus | 22 | -88 | -4 |  | -4.3196 | 136 |
|  | R Inferior Frontal Gyrus | 48 | 6 | 16 | 44 | -3.6754 | 355 |
|  | R Middle Temporal Gyrus | 56 | -54 | -2 | 37 | -4.0879 | 346 |

Positive/negative z value means increased/decreased functional connectivity between the groups; MNI, Montreal Neurological Institute; BA, Brodmann area; L, left; R, right; PD, patients with Parkinson's disease; NMC, non-manifesting LRRK2 G2385R and R1628P mutations carriers; NMNC, non-manifesting non-carriers.
